# Multi-Institutional Registry for Prostate Cancer Radiosurgery: A Prospective Observational Clinical Trial

**DOI:** 10.3389/fonc.2014.00369

**Published:** 2015-01-22

**Authors:** Debra Freeman, Gregg Dickerson, Mark Perman

**Affiliations:** ^1^Naples Radiation Oncology, Naples, FL, USA; ^2^Anova Cancer Center, Denver, CO, USA; ^3^South Florida Radiation Oncology, Stuart, FL, USA

**Keywords:** prostate radiosurgery stereotactic, prostate SBRT, observational research, hypofractionated stereotactic radiation, CyberKnife robotic radiosurgery

## Abstract

**Objective:** To report on the design, methodology, and early outcome results of a multi-institutional registry study of prostate cancer radiosurgery.

**Methods:** The Registry for Prostate Cancer Radiosurgery (RPCR) was established in 2010 to further evaluate the efficacy and toxicity of prostate radiosurgery (SBRT) for the treatment of clinically localized prostate cancer. Men with prostate cancer were asked to voluntarily participate in the registry. Demographic, baseline medical, and treatment-related data were collected and stored electronically in a Health Insurance Portability and Accountability Act-compliant database, maintained by Advertek, Inc. Enrolled men were asked to complete short, multiple choice questionnaires regarding their bowel, bladder, and sexual function. Patient-reported outcome forms were collected at baseline and at regular intervals (every 3–6 months) following treatment. Serial prostate-specific antigen measurements were obtained at each visit and included in the collected data.

**Results:** From July 2010 to July 2013, nearly 2000 men from 45 participating sites were enrolled in the registry. The majority (86%) received radiosurgery as monotherapy. At 2 years follow-up, biochemical disease-free survival was 92%. No Grade 3 late urinary toxicity was reported. One patient developed Grade 3 gastrointestinal toxicity (rectal bleeding). Erectile function was preserved in 80% of men <70 years old. Overall compliance with data entry was 64%.

**Conclusion:** Stereotactic radiosurgery is an alternative option to conventional radiotherapy for the treatment of organ-confined prostate cancer. The RPCR represents the collective experience of multiple institutions, including community-based cancer centers, with outcome results in keeping with published, prospective trials of prostate SBRT.

## Background

The feasibility of image-guided robotic radiosurgery for treating localized prostate cancer was first described by King at Stanford University (1). His phase I protocol delivered 36.25 Gy in five fractions of 7.25 Gy to the prostate using a CyberKnife treatment platform. He initially reported on acute and 18-month late toxicity in 26 low-risk patients. No patient experienced grade 3 or 4 acute or late toxicity, and only one patient experienced grade 2 late morbidity (urethral stricture). The mean prostate-specific antigen (PSA) at 18 months after treatment was 0.22 ng/ml. This was followed by a prospective Phase II clinical trial for 41 low-risk prostate cancer patients with 6 months’ minimum follow-up (1). The early (<3 months) and late (>6 months) urinary and rectal toxicities were assessed using validated quality of life questionnaires including the International Prostate Symptom Score (IPSS), Expanded Prostate Cancer Index Composite score and the Radiation Therapy Oncology Group (RTOG) toxicity criteria. Patterns of PSA response were also analyzed. At a median follow-up of 33 months, there were no RTOG Grade 4 acute or late rectal/urinary complications. There were two patients with RTOG Grade 3 late urinary toxicity and none with RTOG Grade 3 rectal complications. At last follow-up, no patient has had a PSA failure. Of 32 patients with 12 months minimum follow-up, 25 patients (78%) achieved a PSA nadir 0.4 ng/ml. A PSA decline to progressively lower nadirs up to 3 years after treatment was observed.

The Multi-institutional Registry for Prostate Cancer Radiosurgery (RPCR, Inc.), a not-for-profit, 501c-6 organization, was established in 2010 to further evaluate the efficacy and toxicity of stereotactic radiosurgery in the treatment of prostate cancer. RPCR, Inc. originated as a collaborative effort between the Florida Robotic Radiosurgery Association (now closed) and several community-based radiosurgery centers in Florida. The initial goal was to collect data on prostate cancer patients treated with hypofractionated, stereotactically delivered radiation at non-academic radiosurgery centers in Florida, and share this “real world” experience with the Centers for Medicare and Medicaid Services (CMS) and the Medicare Administrative Contractor for Florida, First Coast Service Options.

RPCR, Inc. launched the registry in July 2010, and by the end of the first year, more than 300 patients had been enrolled. Radiosurgery centers outside of Florida became interested in having a data collection tool for their prostate cancer patients, and the once Florida-based project was gradually expanded to its current form: a multi-state, multi-institutional database specifically for prostate cancer patients treated with hypofractionated radiation using stereotactic techniques.

## Methods

The original platform for the RPCR registry database was modeled on a pre-existing registry database managed by the former CyberKnife Society, now the Radiosurgical Society (RSS). That registry, now known as the RSSearch™ Registry, was designed to collect data on SRS and SBRT treatment practices and outcomes for all disease sites and help establish “best practice” guidelines for SRS/SBRT delivery (2). Both the RPCR and RSSearch™ registries are housed and maintained by Advertek, Inc., a medical software company from Louisville, KY, USA. Founded in 1999, Advertek focuses on software and internet-based applications for the management of research data, patient documentation, statistical analysis, and medical web communications.

Since the RPCR registry was conceived to address the concerns of CMS and First Coast Service Options regarding coverage of prostate radiosurgery, RPCR’s development team borrowed data fields created for the prostate sub-section of the RSSearch™ Registry and re-structured them to focus on the data elements important to third-party payers, namely disease-specific outcomes and quality of life measures. In addition, data fields were streamlined to facilitate data entry by community-based oncology practices, most of whom do not have the infrastructure to support a dedicated research team. RPCR, Inc. contracted with Advertek, Inc. to house and maintain the patient data. Advertek insures that the RPCR registry database strictly complies with all Health Insurance Portability and Accountability Act (HIPAA) requirements for system security and privacy. Access to individual patient data is restricted to authorized, site-specific users, and aggregate data analyses are conducted not by RPCR users but by independent contractors with access to only selected patient information.

The RPCR registry database is divided into three main sections, or “forms”: screening, treatment, and follow-up. Each new patient enrolled in the RPCR registry is assigned a unique registry identification number by Advertek, and participating sites are responsible for linking this number with the appropriate patient at their site. No patient names or initials are recorded in the database. The only persons or organizations able to see identifying information are select staff at the patient’s local treatment facility and the independent database vendor (Advertek). No other participant in the RPCR registry has access to these unique identification numbers.

Information captured in the screening form includes referral source, third-party payer, radiation delivery device, patient age, Karnofsky performance status, rationale for radiosurgery, initial TNM stage, Gleason score, number of positive biopsy cores, use of hormonal therapy, and several baseline measures, including pre-treatment PSA, IPSS, International Index of Erectile Function (IIEF-5) score, Bowel Health Inventory score, and Visual Analog pain score, the last of which CMS requested be added.

The treatment form selected by a participating site depends on which technical device is used to deliver the radiosurgery, but all capture date of initial treatment, date of treatment completion, dose calculation method, plan type (isocentric vs. non-isocentric), number of fractions, number of active beams, tracking method used (fiducials vs. cone beam CT), collimator type (fixed vs. dynamic), prescribed dose, delivered dose, total monitor units, and maximum dose delivered. Doses to specific organs at risk, including rectum, bladder, penile bulb, and testicles, are also recorded.

The follow-up form repeats many of the elements of the initial screening form, including performance status, pain score, patient-reported outcome scores, and post-treatment PSA. It also includes physician assessment of potential treatment-related toxicity, using Common Toxicity Criteria for Adverse Event Reporting, version 3 (CTCAE v3). Participating sites may obtain follow-up information as often as they choose, but RPCR encourages sites to record follow-up data every 3 months for the first 2 years following radiosurgical treatment and every 6–12 months thereafter, for a minimum of 5 years.

Each treatment facility (“participating site”) in the RPCR registry is required to sign a letter of agreement with RPCR, Inc. prior to enrolling patients in the database. Sites are informed that they will be responsible for entering their own data into the database, and each site is responsible for the integrity of the data entered. RPCR, Inc. cannot validate the accuracy of data entered by any participating site. However, quality assurance measures are built into the system to reduce errors in electronic data entry, and staffs at each participating site are provided with individualized training on the web-based system by Advertek, prior to entering patient data. While participating sites have open access to their own data, they are restricted from access to any other site’s information. Aggregate data analyses for benchmark comparisons are available upon request. Any participating site may elect to exclude its own data from aggregate reporting. In such cases, however, the participating site may not request aggregate data, other than what is available through published outcome reports.

Patient participation in the RPCR registry is, of course, entirely voluntary, but patients are required to read and sign an informed consent prior to being enrolled in the registry. The informed consent advises them that they will be asked to complete short questionnaires prior to and periodically after treatment, and answer multiple choice questions about their bowel, bladder, and sexual functioning. They are informed that their medical information will be protected and secure, but information related to their condition and treatment may be used for aggregate data analysis. They are specifically informed that participation in the registry is not required for them to receive treatment for their prostate cancer.

## Results

Since launching in July 2010, the RPCR registry has enrolled over 2700 men from 45 participating sites, including academic centers, hospital-based practices, and free-standing centers. All enrolled patients received stereotactic radiosurgery as at least one component of their overall treatment for prostate cancer. The present report will focus on the nearly 2000 patients enrolled in the registry between July 2010 and July 2013.

**Figure 1:**
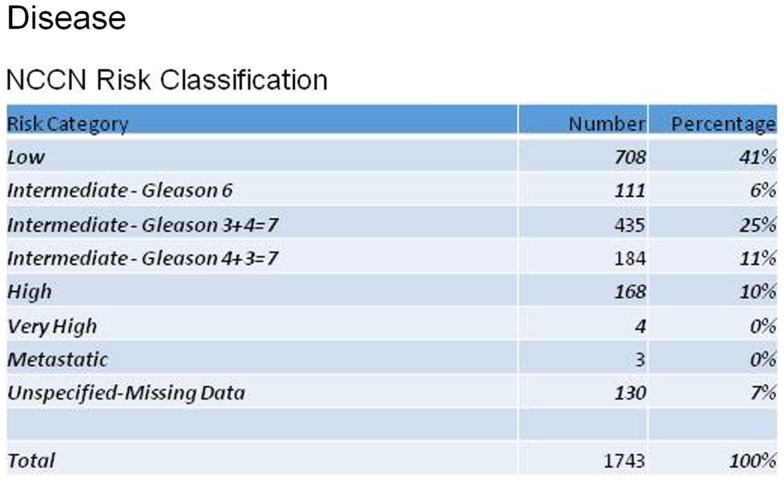
**Patient stratification according to NCCN Risk Classifications for prostate cancer**.

Reviewing patient characteristics, the mean age of enrolled men was 68 years (43–100). The majority (77%) were Caucasian, with 11% African-American. Men were referred for radiosurgery primarily by their urologists (69%), but 18% were self-referrals. Figure 1 shows the distribution of patients according to the NCCN recurrence risk classification for prostate cancer, based on AJCC stage, Gleason score, and pre-treatment PSA. Mean initial, pre-treatment PSA for all patients was 6.7 ng/ml.

Treatment forms were completed for the majority of enrolled patients (89%). For those with recorded data, 86% were treated using radiosurgery as monotherapy, with doses ranging from 35 to 40 Gy in four to five fractions. A small percentage (8%) received radiosurgery as a “boost” following 45–50 Gy of external beam radiation, with doses of 19.5–21.75 Gy in three fractions. All patients in this report were treated on CyberKnife Robotic Radiosurgery(R) delivery systems using fiducial tracking algorithms.

**Figure 2:**
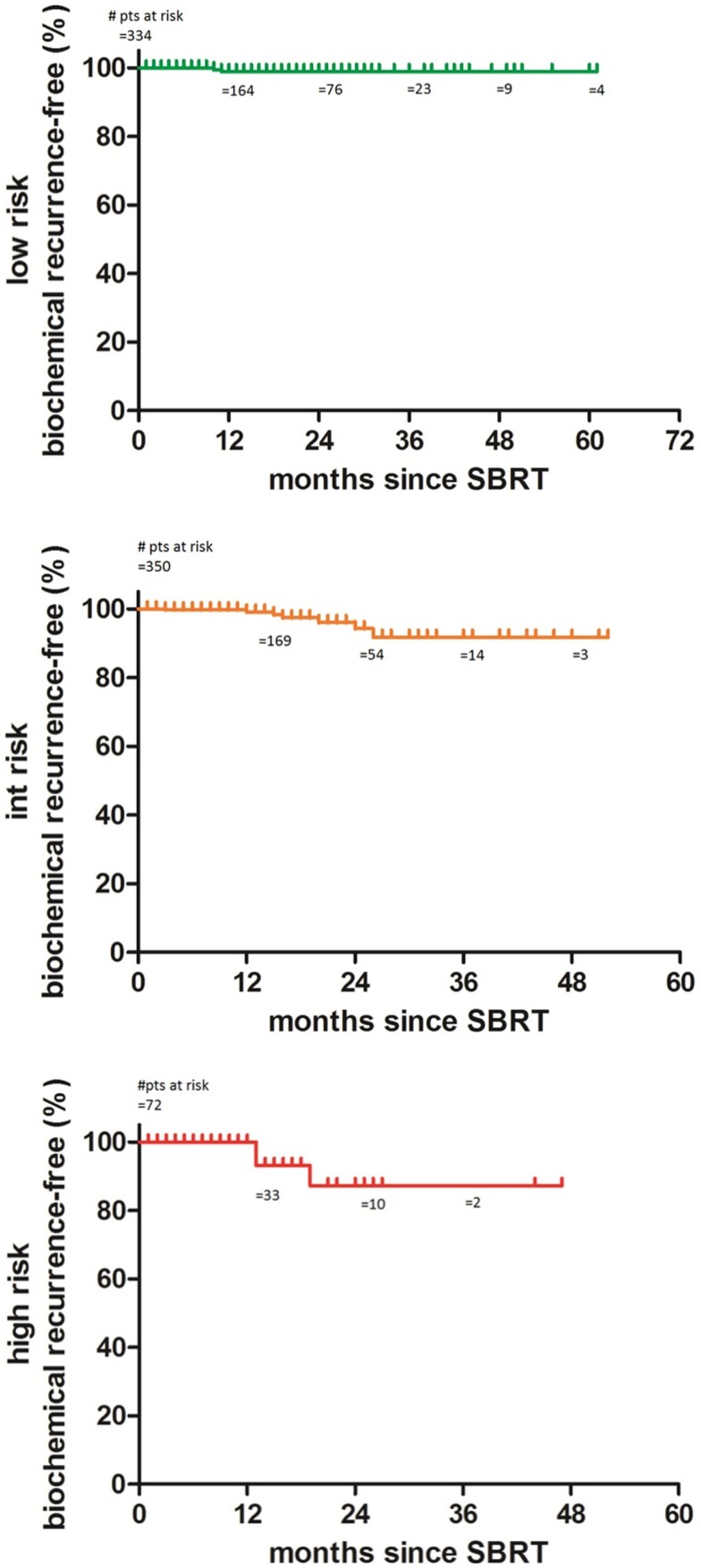
**Actuarial biochemical disease-free survival, stratified by risk group**.

**Figure 3:**
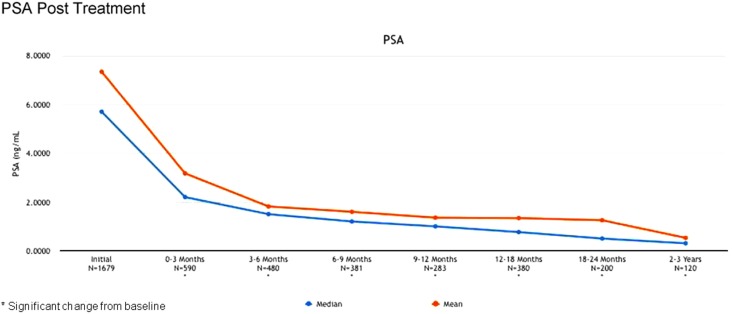
**Actuarial PSA response following treatment**.

The 2-year biochemical disease-free survival (bDFS) for the entire patient cohort was 92%. Stratified by risk group, 2-year bDFS was 99, 97, 85, and 87% for low risk, intermediate risk (Gleason 3 + 4), intermediate risk (Gleason 4 + 3) and high risk patients, respectively (*p* = 0.03) (Figure 2). PSA levels declined sharply in the first 6 months following radiosurgery, with a continued downward trend in PSA levels between 18 months and 2 years following treatment (Figure 3).

Twenty-two patients experienced a rise in PSA after treatment of more than 2 ng/ml, following a nadir level (Phoenix definition of biochemical recurrence). However, only six of these patients had biopsy-proven or radiographic confirmation of disease recurrence. Nine experienced a temporary “bounce” in PSA level that subsequently declined, with no documented evidence of disease recurrence. This PSA bounce phenomenon typically occurred between 12 and 18 months following treatment, similar to the reported experience following external beam radiation (3).

### Toxicity

Acute (0–3 months) and late (3–24 months) toxicity following prostate radiosurgery was assessed using both patient-reported outcome measures and physician evaluations. The IPSS was used for patient-reported genitourinary symptoms. The International Index of Erectile Function (IIEF-5) was used for evaluation of sexual function post-treatment. The Bowel Health Inventory for men was used for patient-reported gastrointestinal symptoms. During the first 3 months following treatment, the most commonly reported acute toxicities were Grade 1 urinary symptoms, including urgency, frequency, and dysuria. IPSS scores increased from a mean baseline of 7 to a mean score of 10 between 0 and 3 months. A similar uptick was *not* noted for Bowel Health Inventory scores in the first 3 months following treatment.

**Figure 4:**
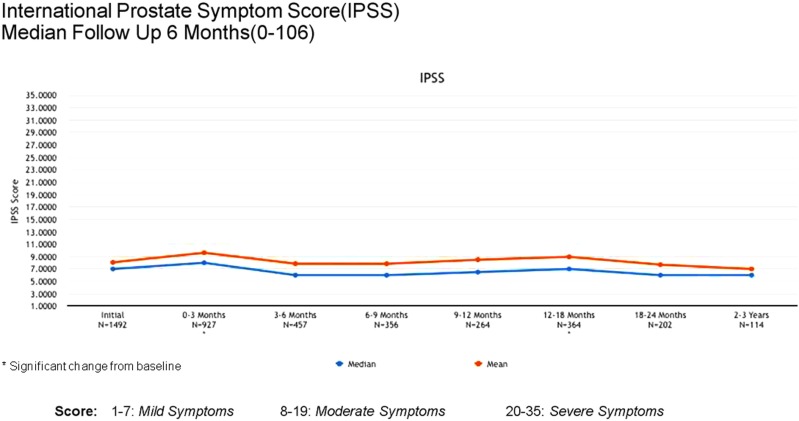
**IPSS scores at baseline and following treatment**.

**Figure 5:**
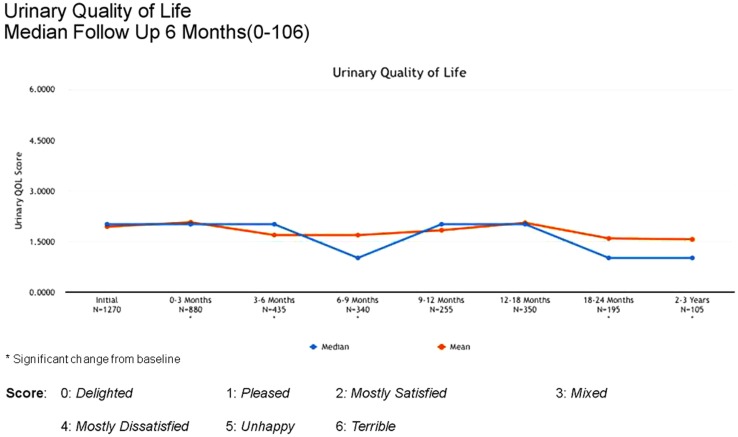
**Urinary quality of life following treatment**.

**Figure 6:**
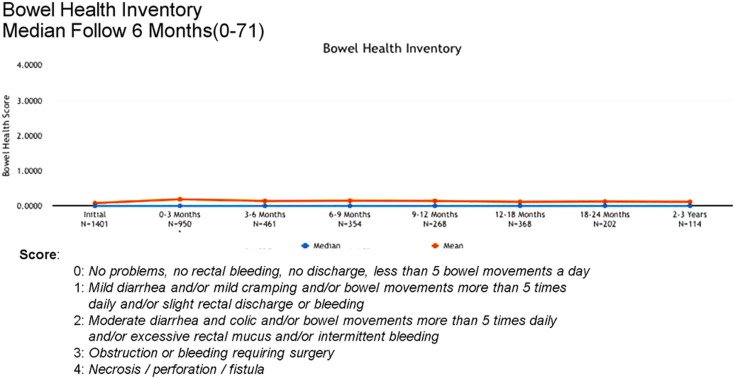
**Bowel Health Inventory scores at baseline and following treatment**.

Between 3 and 24 months, only one Grade 3 gastrointestinal toxicity was reported (rectal bleeding). No Grade 3 late genitourinary toxicities have been recorded. Approximately 10% of patients reported mild urinary symptoms persisting more than 3 months following treatment. However, IPSS scores returned to baseline levels after the initial slight increase and remained stable. Urinary quality of life scores also remained stable post-treatment (Figures 4–6).

**Figure 7:**
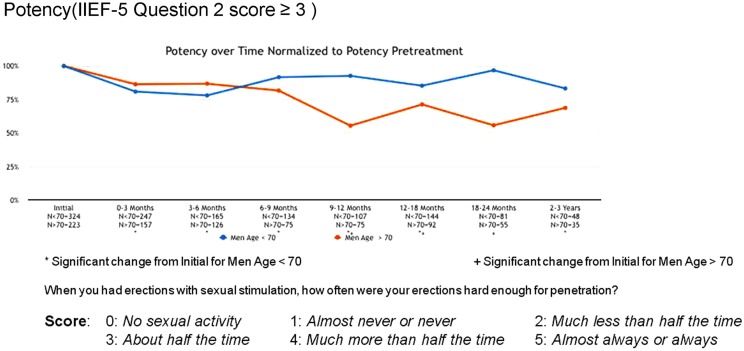
**Erectile function (potency) following treatment**.

Erectile function scores did show an overall downward trend following treatment. When stratified by age, men younger than 70 years demonstrated higher baseline IIEF-5 scores (median 20 vs. 12) and maintained higher post-treatment scores (median 17 vs. 5) than men older than 70. Approximately 80% of men <70 maintained erections sufficient for intercourse following radiosurgery, while only 55% of men >70 did so (Figure 7).

## Discussion

Several clinical studies supporting the safety and efficacy of hypofractionated, stereotactic radiosurgery in the management of prostate cancer have now been published. Freeman and King reported 5-year clinical outcome results in 41 low-risk patients from their combined institutions (4). At a median of 60 months, the biochemical disease-free survival (DFS) was 93%. No Grade 3 rectal toxicity was noted. A single patient developed Grade 3 urinary toxicity. No biochemical failures were noted after 42 months. Katz has also published 5-year outcome results on 304 patients with low and intermediate disease treated with CyberKnife radiosurgery (5). Biochemical DFS was 96%; no significant toxicity was reported.

The largest series to date is that from King et al. (6), who reported pooled outcome results from eight institutions on 1100 patients with localized prostate adenocarcinoma, treated with stereotactically delivered radiation. A subset of 135 patients had a minimum of 5 years follow-up. The actuarial bDFS at 5 years was 97% for low-risk patients. For intermediate/high risk patients, bDFS was 90%. No grade 3 toxicity was reported. Katz recently published 6-year outcome data on 300+ low- and intermediate-risk patients from a single institution, with similar results (7). The prescribed dose per fraction in both of these series was >5 Gy, which is now commonly referred to as “extreme” hypofractionation.

In 2012, the American Society of Therapeutic Radiation Oncology (ASTRO) released a revised position statement on the use of radiosurgery in the management of prostate cancer. “It is ASTRO’s opinion that data supporting the use of SBRT for prostate cancer have matured to a point where SBRT could be considered as an appropriate alternative for select patients with low to intermediate-risk disease” (8). The National Cancer Care Network guidelines, version 2.2014, now include radiosurgery as a treatment alternative for low-intermediate-risk prostate cancer patients. “Extremely hypofractionated image-guided IMRT/SBRT regimens are an emerging treatment modality, with single institution and pooled reports of similar efficacy and toxicity to conventionally fractionated regimens. They can be considered as a cautious alternative to conventionally fractionated regimens at clinics with appropriate technology, physics, and clinical expertise” (9).

When the RPCR registry was conceptualized in 2009, however, much of this data had not yet been published. The first patient treated in the U.S. with “extremely hypofractionated” radiosurgery for prostate cancer was at Stanford Hospital in 2003. A few community-based centers opened single institution, prospective protocols for prostate radiosurgery shortly thereafter, including Naples Hospital (Freeman and Friedland) in 2005 and Winthrop Hospital (Katz) in 2006. Accuray, Inc. launched two industry-sponsored, multi-center prospective trials for CyberKnife prostate SBRT in 2008, one using homogeneous dose distributions and the other heterogeneous dose distributions, but patient accrual was not completed until 2012, and initial results from these trials have only recently been published (10).

Prior to 2009, the lack of published outcome data on prostate radiosurgery made it understandably difficult for CMS and other third-party payers to define coverage guidelines for this “new” treatment modality for prostate cancer. Submitted claims to Medicare for prostate radiosurgery were handled on a case by case basis. Some regional Medicare Administrative Contractors (MAC’s) developed coverage policies that proposed non-coverage of prostate radiosurgery. In the fall of 2009, First Coast Service Options, the MAC for Florida, released a Local Coverage Determination (LCD) for stereotactic radiosurgery and stereotactic body radiotherapy. (An LCD is a decision by a MAC regarding whether to cover a particular service and whether the service is reasonable and necessary.) Prostate cancer was not included in the list of covered diagnoses for SBRT, based on the lack of long term, published outcome data. However, “favorable consideration” would be given to prostate cancer patients receiving SBRT who were enrolled in a clinical trial^1^.

In April, 2010, the Medicare Evidence Development and Coverage Advisory Committee (MEDCAC) convened in Baltimore, MD, USA to review the clinical evidence for radiation therapy in the management of localized prostate cancer. The committee determined that data on the comparative effectiveness between different forms of radiation treatments, including 3D conformal therapy, IMRT, low dose rate and high dose rate brachytherapy, proton therapy, and SBRT, were inconclusive as to whether one form of radiation therapy was superior to another in terms of overall or disease-specific survival. An apparent “evidence gap” existed. Discussion among many panelists and presenters identified registries, particularly research-based, observational clinical registries such as the RPCR registry, as one method of “bridging” this evidence gap.

In simplest terms, an observational registry is a “collection of selected information about a group of patients who share a common condition or experience” (11). As research tools, registries have been criticized for lack of standardization in data collection, limitations in patient participation due to educational barriers, and the questionable reliability of self-reported data That said, registries can provide valuable information for both clinicians and patients. Observational registry outcome data paved the way for MammoSite™ accelerated partial breast radiation to be accepted in clinical radiation oncology practice. The American Society of Breast Surgeons MammoSite Breast Brachytherapy Registry Trial launched in 2002 in multiple centers across the United States. Six-year outcome data on 1440 patients were published in 2012, showing low local recurrence rates in the treated breast, comparable to standard, whole-breast radiation, with good/excellent cosmesis in 90% of patients (12). Balloon brachytherapy was subsequently included as one arm of the NSABP/RTOG B39 prospective, randomized clinical trial comparing various partial breast irradiation techniques. Due to the growing popularity of registry databases, the Agency for Healthcare Research and Quality (AHRQ) actually launched a “registry of patient registries” in 2012 to facilitate patient and physician access to these various research efforts (13). The RPCR is among the listed studies.

Has the RPCR registry been successful thus far in bridging the evidence gap for prostate SBRT? Certainly, patient accrual has been better than anticipated when the registry first opened in 2010 with 10 sites in Florida. The number of participating sites continues to grow, in large part due to “favorable consideration” being given to registry participation by most Medicare Administrative Contractors across United States. A stable technical platform, a user-friendly database, and readily available technical support are also significant factors in the registry’s success. The relatively inexpensive enrollment costs ($5000 for 3-year participation) have made the RPCR registry accessible to community-based cancer centers as well as academic centers. Perhaps most importantly, participating sites have a genuine interest in offering quality cancer care to their patients, and many use the registry as a metric to compare their outcome results with those of their colleagues.

Like most registry studies, the RPCR registry faces several ongoing challenges. Compliance with data entry, particularly follow-up data, has been sub-optimal. While nearly 90% of enrolled patients have complete screening and treatment information in the database, only 64% have complete follow-up data. In an effort to improve compliance, RPCR, Inc. recently partnered with VisionTree™, Inc. to develop an online patient-reported outcomes system for the RPCR registry. Using a secure, web-based environment, patients can now complete their required outcome forms (IPSS, IIEF-5, and Bowel Health Inventory) without having to travel to a physician’s office.

Maintaining quality and integrity of the data is another challenge. RPCR, Inc. does not have the resources to support a Data Safety Monitoring Board or Quality Assurance committee. However, all data analyses and compliance reports to date have been generated by independent contractors, outside the RPCR registry system, and with restricted access to aggregate data. Any data outlier (a result not within the expected range for a given data field) is reported to RPCR’s compliance officer for validation and/or correction. To maintain interest in the registry’s continued success, RPCR, Inc. will be distributing annual reports of outcome results to all participating sites, expanding the database to include a variety of treatment platforms, and continuing to collaborate with colleagues from the RSS, Cyberknife Coalition, and CMS.

## Conclusion

Registry for Prostate Cancer Radiosurgery, Inc. started as a grassroots effort by a few physicians with a common goal: to bring prostate radiosurgery to the mainstream of treatment options for patients with prostate cancer. Four years, 45 sites, and 2700 patients later, the RPCR registry has become a robust clinical database, with outcome results comparable to those from several prospective clinical trials. The RPCR registry represents “real world” experience, and offers non-academic, community-based cancer centers the opportunity to contribute their patient data to a growing body of clinical knowledge about prostate radiosurgery. As the data continue to mature and mechanisms for capturing follow-up data improve, we look forward to sharing long-term outcome results from the RPCR registry database.

## Conflict of Interest Statement

Debra Freeman, MD was previously employed by Accuray, Inc. in the Dept. of Medical Affairs from August 2008 to January 2010 and served as a medical consultant until January 2012. Drs. Dickerson and Perman have no competing interests.

## Footnotes

^1^Medicare Part B Local Coverage Determination (LCD) Comment Summary. (2009, Draft comments 155452).
